# Endothelial *Adenosine Monophosphate-Activated Protein Kinase-Alpha1* Deficiency Potentiates Hyperoxia-Induced Experimental Bronchopulmonary Dysplasia and Pulmonary Hypertension

**DOI:** 10.3390/antiox10121913

**Published:** 2021-11-29

**Authors:** Ahmed Elsaie, Renuka T. Menon, Amrit K. Shrestha, Sharada H. Gowda, Nidhy P. Varghese, Roberto J. Barrios, Cynthia L. Blanco, Girija G. Konduri, Binoy Shivanna

**Affiliations:** 1 Department of Pediatrics, Neonatology Section, Baylor College of Medicine (BCM), Houston, TX 77030, USA; aelsaie@cmh.edu (A.E.); Renuka.Menon@bcm.edu (R.T.M.); Amrit.Shrestha@bcm.edu (A.K.S.); Sharada.Gowda@bcm.edu (S.H.G.); 2 Department of Pediatrics, Cairo University, Cairo 11956, Egypt; 3 Department of Pediatrics, Pulmonology Section, Baylor College of Medicine (BCM), Houston, TX 77030, USA; npvarghe@texaschildrens.org; 4 Department of Pathology, Houston Methodist Hospital, Houston, TX 77030, USA; rbarrios@houstonmethodist.org; 5 Department of Pediatrics, Division of Neonatology, University of Texas Health Science Center, San Antonio, TX 78229, USA; Blanco@uthscsa.edu; 6 Department of Pediatrics, Division of Neonatology, Children’s Research Institute, Medical College of Wisconsin, Milwaukee, WI 53226, USA; gkonduri@mcw.edu

**Keywords:** bronchopulmonary dysplasia, pulmonary hypertension, hyperoxia, *AMPKα1*, neonatal HPMECs

## Abstract

Bronchopulmonary dysplasia and pulmonary hypertension, or BPD-PH, are serious chronic lung disorders of prematurity, without curative therapies. Hyperoxia, a known causative factor of BPD-PH, activates adenosine monophosphate-activated protein kinase (AMPK) α1 in neonatal murine lungs; however, whether this phenomenon potentiates or mitigates lung injury is unclear. Thus, we hypothesized that (1) endothelial *AMPKα1* is necessary to protect neonatal mice against hyperoxia-induced BPD-PH, and (2) *AMPKα1* knockdown decreases angiogenesis in hyperoxia-exposed neonatal human pulmonary microvascular endothelial cells (HPMECs). We performed lung morphometric and echocardiographic studies on postnatal day (P) 28 on endothelial *AMPKα1*-sufficient and -deficient mice exposed to 21% O_2_ (normoxia) or 70% O_2_ (hyperoxia) from P1–P14. We also performed tubule formation assays on control- or *AMPKα1*-siRNA transfected HPMECs, exposed to 21% O_2_ or 70% O_2_ for 48 h. Hyperoxia-mediated alveolar and pulmonary vascular simplification, pulmonary vascular remodeling, and PH were significantly amplified in endothelial *AMPKα1*-deficient mice. *AMPKα1* siRNA knocked down *AMPKα1* expression in HPMECs, and decreased their ability to form tubules in normoxia and hyperoxia. Furthermore, *AMPKα1* knockdown decreased proliferating cell nuclear antigen expression in hyperoxic conditions. Our results indicate that *AMPKα1* is required to reduce hyperoxia-induced BPD-PH burden in neonatal mice, and promotes angiogenesis in HPMECs to limit lung injury.

## 1. Introduction

Bronchopulmonary dysplasia (BPD) remains the most common adverse outcome in preterm neonates, and is still one of the most challenging complications in perinatal medicine. With the improved survival of extremely premature infants, the incidence of BPD remains high, at about 30%, depending on the cohort and the definition used [1,2,3,4]. Pulmonary hypertension (PH) is one of the most serious long-term morbidities of BPD. PH develops in 17–24% of BPD patients [5,6,7], and is associated with poor clinical outcomes manifested by increased mortality rates [5] and adverse neurodevelopmental sequelae [8].

The histopathology of BPD-associated PH (BPD-PH) is characterized by fewer enlarged alveoli with decreased septation (alveolar simplification), and fewer, dysmorphic lung capillaries (pulmonary vascular simplification) [9]. Disrupted lung angiogenesis is a hallmark of BPD [10]. Lung angiogenesis is continuously involved in the process of alveolarization, and disrupted angiogenesis leads to abnormal alveolarization [11]. The major pathogenic factors of BPD-PH are decreased growth and function and altered vasoreactivity and extracellular matrix of lung endothelial cells [11]. However, targeted therapies for this disorder remain elusive, because the molecular mechanisms regulating these pathogenic processes are poorly understood. Molecular targets that facilitate the development and function of the lung vasculature are vital to decrease this disease burden.

Adenosine monophosphate-activated protein kinase (AMPK) could be one such molecular target. AMPK is a serine-threonine heterotrimeric protein kinase that has a catalytic α-subunit, a scaffolding β subunit, and a regulatory γ-subunit [12]. The phosphorylation of the catalytic α1/α2-subunit at threonine 172 (Thr 172) activates AMPK [13]. This evolutionarily conserved kinase is known to maintain energy homeostasis [14]; however, evolving evidence suggests an equally important role for AMPK in endothelial biology [15]. AMPKα induces proliferation and facilitates the growth and survival of endothelial cells, while promoting apoptosis in vascular smooth muscle cells [15,16,17,18]. Additionally, we observed that hyperoxia exposure, a major causative factor of BPD-PH, increases AMPKα activation in neonatal murine lungs [19]. The altered expression of AMPKα in hyperoxia-exposed developing lungs indicates that *AMPKα* plays a role in lung development, injury, and repair. The role of AMPK in experimental lung injury and PH has been previously investigated [15,20,21]. However, several factors remain poorly understood, including the effects of endothelial *AMPKα1* signaling on lung and heart function in neonatal hyperoxic injury, and our studies were designed to address these gaps.

The oxidative stress and inflammation associated with oxygen therapy are established risk factors for the development of BPD-PH. Since the lung developmental stages of mice and extremely preterm infants are similar at birth [22], and the lung phenotype of hyperoxia exposed neonatal mice resembles human BPD-PH [23,24,25,26,27], we decided to use this model to test the hypothesis that endothelial *AMPKα1* is necessary to decrease hyperoxia-induced experimental BPD-PH burden in neonatal mice. In addition, we used neonatal human pulmonary microvascular endothelial cells (HPMECs) to elucidate *AMPKα1′*s role in human lung angiogenesis, and enhance the translational potential of our murine studies. HPMECs were chosen because these cells express *AMPKα1* and are commonly used to decipher the mechanistic and therapeutic roles of several molecules in the lung endothelium [15,28,29,30].

## 2. Materials and Methods

### 2.1. In Vivo Studies

#### 2.1.1. Animals

This study was approved by the Institutional Animal Care and Use Committee of Baylor College of Medicine (Protocol # AN-5631). *AMPKα1^flox^*^/*flox*^ and *Tie2*-Cre mice on a C57BL/6J background and C57BL/6J wild-type (WT) mice were obtained from the Jackson Laboratory (Bar Harbor). Generation of endothelial AMPKα1-deficient mice: To disrupt lung endothelial *AMPKα1* signaling, the endothelial *AMPKα1* expression was reduced by mating *AMPKα1^flox^*^/*flox*^ with *Tie2-Cre* mice. We validated the genotype by genotyping and immunoblotting analysis of the lung tissues for *AMPKα1* expression.

#### 2.1.2. Hyperoxia Experiments

Male and female endothelial *AMPKα1*-sufficient (*eAMPKα1*^+/+^) or -deficient (*eAMPKα1*^+/−^) mice were continuously exposed to 21% O_2_ (normoxia) or 70% O_2_ (hyperoxia) from P1–P14, as described before [31].

#### 2.1.3. Lung Endothelial Cell Extraction and Immunoblot Assays

Endothelial cells from the lungs of experimental animals were extracted, as described previously [32]. Briefly, lung tissues were minced in petri dishes containing Roswell Park Memorial Institute (RPMI) 1640 medium supplemented with 10% fetal calf serum, penicillin/streptomycin, 10 mM 4-(2-hydroxyethyl)-1-piperazineethanesulfonic acid (HEPES), 20 mM L-glutamine, 5 mg/mL of type I collagenase, and 1 mg/mL of Type I DNase. Collagenase digestion was performed for 30 min at 37 °C on a rotary agitator, at a speed of 125 RPM. Any remaining undigested tissue was mechanically disrupted by passing through an 18 G needle attached to a 5-mL syringe three times, followed by passage through a 70 mm cell strainer. The cells were then centrifuged at 400 g for 5 min at 4 °C. The supernatant was discarded, and the red blood cells (RBCs) in the cell pellet were lysed using RBC lysis buffer, following which, the cell pellet was harvested by centrifugation at 400 g for 5 min at 4 °C, and discarding the supernatant. The cell pellet was resuspended in PBS containing 0.1% BSA, and incubated with anti-platelet and endothelial cell adhesion molecule 1 (PECAM-1) antibody-conjugated Dynabeads from Life Technologies (Carlsbad, CA, USA) on a rocker for 30 min at room temperature, as per the manufacturer’s instructions. After isolation, cells were washed with PBS three times, and the protein was extracted using a lysis buffer (Santa Cruz Biotechnologies, Santa Cruz, CA, USA; sc-24948), as per the manufacturer’s recommendations. The lung endothelial cell protein lysates were then subjected to immunoblotting using antibodies against: *AMPKα1* (Abcam, Cambridge, UK; ab3759), *AMPKα2* (Abcam; ab3760), and glyceraldehyde 3-phosphate dehydrogenase ([GAPDH] Cell Signaling, Danvers, MA, USA; 2118).

#### 2.1.4. Analysis of Alveolarization and Pulmonary Vascularization

The alveolarization was evaluated on P28 by quantifying mean linear intercepts (MLI) and the radial alveolar counts (RAC), as described before [26]. Lung vascular development was also determined on P28 by quantifying vWF-stained lung blood vessels with a diameter of <150 µm [31].

#### 2.1.5. Pulmonary Vascular Remodeling

Pulmonary vascular remodeling was evaluated by quantifying the medial thickness index of resistance pulmonary blood vessels. Deparaffinized lung tissues were subjected to immunostaining using α-smooth muscle actin (α-SMA) antibody (Sigma-Aldrich, St. Louis, MO, USA; A5228), and the medial thickness index was estimated using the equation: [(areaext − areaint)/areaext] × 100, where areaext and areaint represent the areas within the external and internal borders of the α-SMA layer, respectively [23].

#### 2.1.6. Echocardiography

Transthoracic echocardiography was performed on P28, to evaluate the indices of PH, as described previously [26]. Pulsed-wave Doppler recording of the pulmonary blood flow obtained at the aortic valve level in the parasternal right ventricular outflow view was used to estimate pulmonary acceleration time (PAT) [33]. The right ventricular systolic pressure (RVSP) was estimated by the regression formula RVSP = 63.7 − (1.5 × PAT) [33].

#### 2.1.7. Estimation of the Right Ventricle (RV)/Left Ventricle (LV) Free Wall Thickness Ratio

Hematoxylin and eosin-stained sections of paraffin-embedded heart were used to analyze RV/LV free wall thickness ratio, as we have described recently [34].

### 2.2. In Vitro Studies

#### 2.2.1. Cell Culture

The neonatal human pulmonary microvascular endothelial-like cells (HPMECs) were obtained from the American Type Culture Collection (ATCC^®^ CRL-3244). We grew these cells based on the manufacturer’s protocol, and used cells between passages, five and eight, for our studies.

#### 2.2.2. Small Interfering RNA (siRNA) Transfection Experiments

We performed transient transfections with either 50 nM control siRNA (Dharmacon, Lafayette, CO, USA; D-001810) or 50 nM target gene-specific *AMPKα1* siRNA (Dharmacon; L-005027), using Lipofectamine RNAiMAX (Life Technologies; 13778030). siRNA-mediated *AMPKα1* knockdown was confirmed by RT- PCR analysis and immunoblotting.

#### 2.2.3. Hyperoxia Experiments

HPMECs transfected with control (*SiC*) or *AMPKα1* (*SiAMPKα1*) siRNA were exposed to 21% O_2_ + 5% CO_2_ (normoxia) or 70% O_2_ + 5% CO_2_ (hyperoxia) for 48 h, as described previously [35].

#### 2.2.4. Real-Time RT-PCR Assays

We initially checked for the integrity and quality of our RNA by denaturing agarose gel and measuring 260/280 ratio, respectively. Then, we performed real-time quantitative RT-PCR analysis with a 7900HT Real-Time PCR System, using TaqMan gene expression master mix and gene-specific primers (*AMPKα1*-Hs01562315 and *GAPDH*-Hs02758991), as described previously [30]. We used *GAPDH* as the reference gene.

#### 2.2.5. Immunoblot Assays

We performed whole cell protein immunoblotting with the following antibodies: β-actin (Santa Cruz Biotechnologies; sc-47778), *AMPKα1* (Abcam; ab3759), and proliferating cell nuclear antigen (PCNA; Thermo Fisher Scientific, Waltham, MA, USA; MA5-11358).

#### 2.2.6. Tubule Formation Assay

We performed a matrigel assay to determine tubule formation [36]. HPMECs transfected with control or *AMPKα1* siRNA and exposed to normoxia or hyperoxia for 48 h were loaded onto growth factor-reduced matrigel in a 96-well plate. Tubule formation was quantified 24 h later using the Image J software.

#### 2.2.7. Statistical Analysis

Data analysis was done using GraphPad Prism version 9 software (GraphPad Software, La Jolla, CA, USA), and the results are expressed as means ± SD. *p*-value of <0.05 was considered significant. In vivo studies: The effects of exposure, gene, and their associated interactions on outcome variables, including pulmonary alveolarization, pulmonary angiogenesis, and indices of PH, were analyzed using analysis of variance (ANOVA). If a statistical significance of either variable or interaction was noted by ANOVA, the post hoc Bonferroni test was performed. In vitro studies: The effects of hyperoxia, *AMPKα1* knockdown, and their interactions on tubule formation and PCNA expression were analyzed by ANOVA.

## 3. Results

### 3.1. AMPKα1 Deficiency Potentiates Neonatal Hyperoxia-Induced Alveolar and Pulmonary Vascular Simplification in Mice

**Figure 1:**
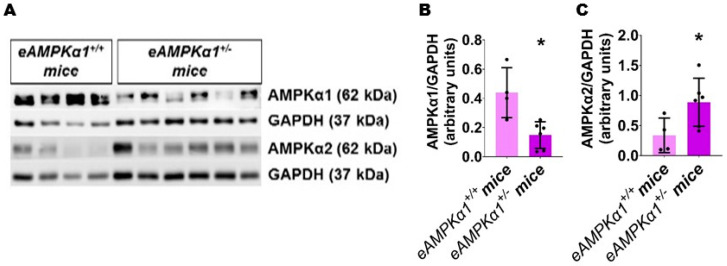
Expression of *AMPKα1* and *AMPKα2* protein in the lung endothelial cells of endothelial *AMPKα1*-sufficient (*eAMPKα1^+/+^)* or -deficient (*eAMPKα1^+/−^)* mice. Lung endothelial cell protein from *eAMPKα1^+/+^* or *eAMPKα1^+/**−**^* mice exposed to 21% O_2_ was harvested on P14 for immunoblot analyses. (**A**–**C**) Immunoblot determination of *AMPKα1*, *AMPKα2*, and GAPDH protein levels (**A**) and quantification and normalization of *AMPKα1* band intensities to GAPDH (**B**) and quantification and normalization of *AMPKα2* band intensities to GAPDH (**C**). Values represent the mean ± SD (*n* = 4–6 mice/genotype). Significant differences between *eAMPKα1^+/+^* and *eAMPKα1^+/**−**^* mice are indicated by *, *p* < 0.05 (*t*-test).

**Figure 2:**
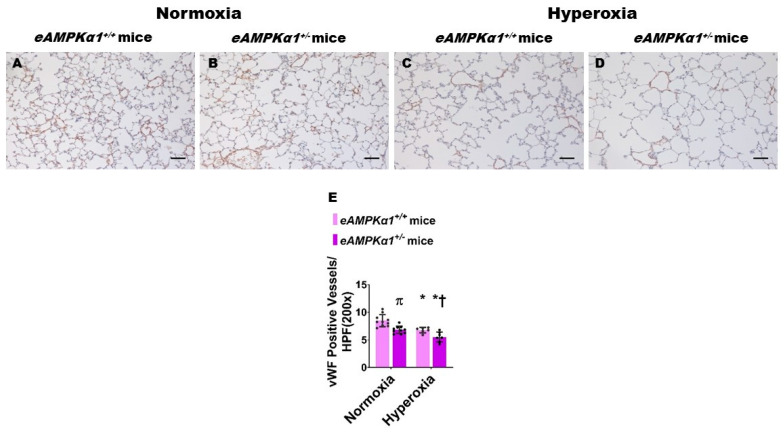
Pulmonary vascularization deficits at postnatal day (P) 28 in endothelial *AMPKα1* deficient (*eAMPKα1^+/−^)* mice exposed to hyperoxia during the first two weeks of life. *eAMPKα1^+/−^* mice and their wild-type littermates (*eAMPKα1^+/+^*) were exposed to either 21% O_2_ (normoxia) for 4 weeks, or 70% O_2_ (hyperoxia) for 2 weeks, followed by normoxia for 2 weeks, and their lung tissues were collected on P28 for lung morphometry studies. (**A**–**D**) Representative vWF-immunostained lung sections from *eAMPKα1^+/+^* (**A**,**C**) or *eAMPKα1^+/−^* (**B**,**D**) mice and exposed to normoxia (**A**,**B**) or hyperoxia (**C**,**D**). Scale bar = 100 µm. (**E**) Pulmonary vascularization was quantified by counting the number of vWF-stained lung blood vessels. Values represent the mean ± SD (*n* = 6–10 mice/group). Significant differences between *eAMPKα1^+/+^* and *eAMPKα1^+/−^* mice are indicated by π, *p* < 0.05 under normoxic conditions and by †, *p* < 0.05 under hyperoxic conditions. Significant differences between the genotype-matched mice under normoxic and hyperoxic conditions are indicated by *, *p* < 0.05. (ANOVA: Effect: *AMPKα1* and hyperoxia, Interaction: No).

**Figure 3:**
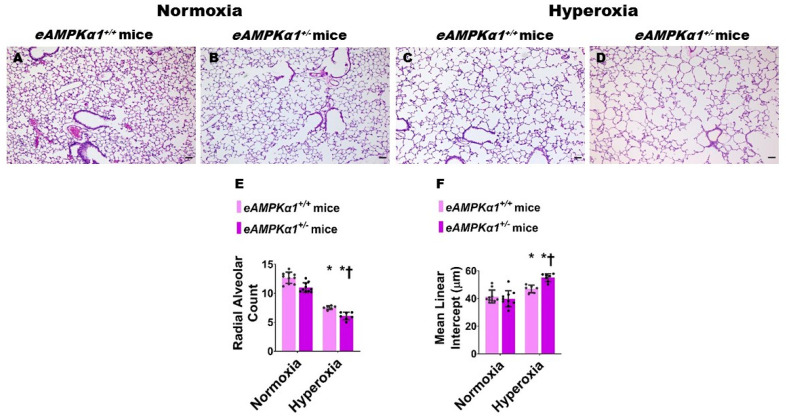
Alveolarization deficits at postnatal day (P) 28 in endothelial *AMPKα1* deficient (*eAMPKα1^+/−^)* mice exposed to hyperoxia during the first two weeks of life. *eAMPKα1^+/−^* mice and their wild-type littermates (*eAMPKα1^+/+^*) were exposed to either 21% O_2_ (normoxia) for 4 weeks, or 70% O_2_ (hyperoxia) for 2 weeks followed by normoxia for 2 weeks, and their lung tissues were collected on P28 to quantify alveolarization. (**A**–**D**) Representative hematoxylin and eosin-stained lung sections from *eAMPKα1^+/+^* (**A**,**C**) or *eAMPKα1^+/**−**^* (**B**,**D**) mice and exposed to normoxia (**A**,**B**) or hyperoxia (**C**,**D**). Scale bar = 100 µm. (**E**,**F**) Alveolarization was quantified by determining radial alveolar count [RAC] (**E**) and mean linear intercept [MLI] **(F**). Values represent the mean ± SD (*n* = 6–10 mice/group). Significant differences between *eAMPKα1^+/+^* and *eAMPKα1^+/−^* mice are indicated by †, *p* < 0.05 under hyperoxic conditions. Significant differences between the genotype-matched mice under normoxic and hyperoxic conditions are indicated by *, *p* < 0.05. (ANOVA: Effect: *AMPKα1* and hyperoxia, Interaction: No).

We identified endothelial *AMPKα1*-deficient and -sufficient mice by genotyping and immunoblotting analysis. The lung endothelial cell *AMPKα1* protein expression was significantly decreased in *eAMPKα1*^+/−^ mice compared with *eAMPKα1*^+/+^ mice at P14 (Figure 1A,B). By contrast, the lung endothelial cell *AMPKα2* protein expression was significantly greater in *eAMPKα1*^+/−^ mice than in *eAMPKα1*^+/+^ mice at P14 (Figure 1A,C). P28 mice exposed to 70% O_2_ (hyperoxia) from P1 to P14 and allowed to recover in 21% O_2_ (normoxia) for 14 days had fewer vWF stained-lung blood vessels than mice who remained in normoxia from P1 to P28 (Figure 2A–E). However, the hyperoxia-induced decrease in vWF stained-lung blood vessels was significantly greater in *eAMPKα1*^+/−^ mice (5.53 ± 0.9) than in *eAMPKα1*^+/+^ mice (6.76 ± 0.48) (Figure 2C–E). Similarly, the lungs of P28 mice exposed to neonatal hyperoxia had fewer and larger alveoli, as evident by decreased RAC and increased MLI, respectively, than the lungs of mice exposed to normoxia (Figure 3A–F). However, the effects of hyperoxia on RAC and MLI were significantly augmented in *eAMPKα1*^+/−^ mice than in *eAMPKα1*^+/+^ mice (Figure 3C–F). These results indicate that endothelial *AMPKα1* deficiency potentiates hyperoxia-induced alveolar and pulmonary vascular simplification.

### 3.2. AMPKα1 Deficiency Potentiates Neonatal Hyperoxia-Induced Pulmonary Vascular Remodeling in Mice

**Figure 4:**
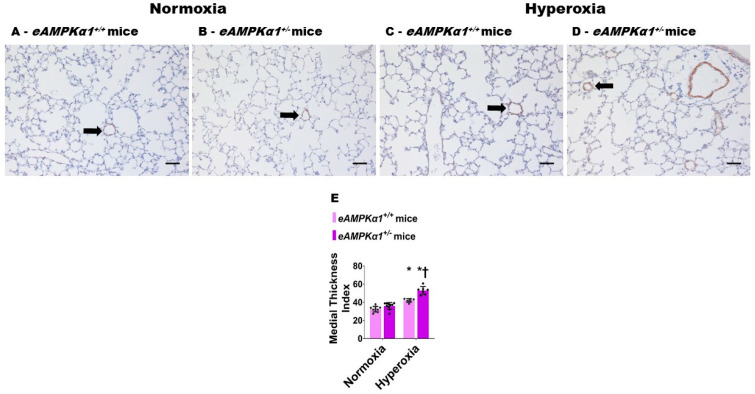
Pulmonary vascular remodeling at postnatal day (P) 28 in endothelial *AMPKα1* deficient (*eAMPKα1^+/−^)* mice exposed to hyperoxia during the first two weeks of life. *eAMPKα1^+/+^* or *eAMPKα1^+/**−**^* mice were exposed to either 21% O_2_ (normoxia) for 4 weeks, or 70% O_2_ (hyperoxia) for 2 weeks, followed by normoxia for 2 weeks, and their lung tissues were collected on P28 for quantifying pulmonary vascular remodeling. (**A**–**D**) Representative alpha-smooth muscle actin (α-SMA) stained blood vessels (arrow) from *eAMPKα1^+/+^* (**A**,**C**) or *eAMPKα1^+/−^* (**B**,**D**) mice, and exposed to normoxia (**A**,**B**) or hyperoxia (**C**,**D**). Scale bar = 100 µm. (**E**) Quantification of pulmonary vascular remodeling by medial thickness index. Values represent the mean ± SD (*n* = 6–10 mice/group). Significant differences between *eAMPKα1^+/+^* and *eAMPKα1^+/−^* mice under hyperoxic conditions are indicated by †, *p* < 0.05. Significant differences between the genotype-matched mice under normoxic and hyperoxic conditions are indicated by *, *p* < 0.05. (ANOVA: Effect: *AMPKα1* and hyperoxia, Interaction: Yes).

P28 mice exposed to neonatal hyperoxia for 14 days showed a significant increase in the medial thickness index of α-SMA stained pulmonary resistance blood vessels (Figure 4C–E). While the medial thickness index of pulmonary resistance blood vessels was similar in *AMPKα1*-deficient and -sufficient mice exposed to normoxia (Figure 4A,B,E), the medial thickness index was significantly greater in *AMPKα1^+/^****^−^*** mice (52.90 ± 4.38) than in *AMPKα1^+/+^* mice (41.96 ± 2.05), when exposed to neonatal hyperoxia (Figure 4C–E), indicating that *AMPKα1* deficiency worsens hyperoxia-induced remodeling of the pulmonary vasculature.

### 3.3. AMPKα1 Deficiency Potentiates Neonatal Hyperoxia-Induced Experimental PH in Mice

**Figure 5:**
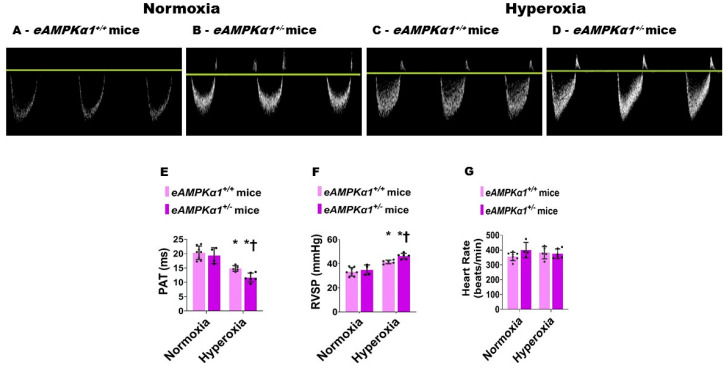
Pulmonary hypertension (PH) indices at postnatal day (P) 28 in endothelial *AMPKα1* deficient (*eAMPKα1^+/−^)* mice exposed to hyperoxia during the first two weeks of life. High-resolution echocardiography was performed on P28 on *eAMPKα1^+/+^* or *eAMPKα1^+/−^* mice exposed to either 21% O_2_ (normoxia) for 4 weeks, or 70% O_2_ (hyperoxia) for 2 weeks followed by normoxia for 2 weeks. (**A**–**D**) Representative Pulsed-wave Doppler (PWD) echocardiography recordings of pulmonary artery blood flow obtained from *eAMPKα1^+/+^* (**A**,**C**) or *eAMPKα1^+/−^* (**B**,**D**) mice and exposed to normoxia (**A**,**B**) or hyperoxia (**C**,**D**). (**E**–**G**) Pulmonary acceleration time [PAT] (**E**), right ventricular systolic pressure [RVSP] (**F**), and heart rate (**G**) were estimated from the PWD pulmonary artery blood flow recordings. Values represent the mean ± SD (*n* = 4–8 mice/group). Significant differences between *eAMPKα1^+/+^* and *eAMPKα1^+/−^* mice under hyperoxic conditions are indicated by †, *p* < 0.05. Significant differences between the genotype-matched mice under normoxic and hyperoxic conditions are indicated by *, *p* < 0.05. (ANOVA: Effect: *AMPKα1* and hyperoxia, Interaction: No).

**Figure 6:**
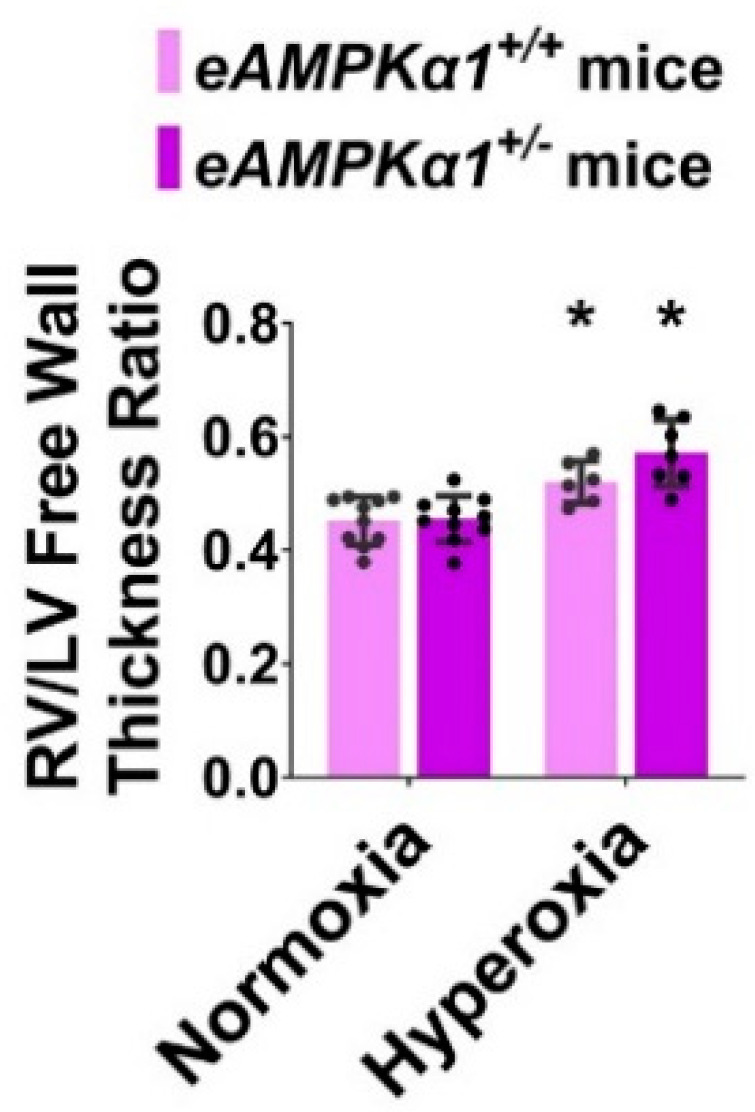
Right ventricle (RV)/left ventricle (LV) free wall thickness ratio at postnatal day (P) 28 in endothelial *AMPKα1*-deficient (*eAMPKα1^+/−^)* mice exposed to hyperoxia during the first two weeks of life. On P28, paraffin-embedded heart sections from *eAMPKα1^+/+^* or *eAMPKα1^+/−^* mice, exposed to either 21% O_2_ (normoxia) for 4 weeks, or 70% O_2_ (hyperoxia) for 2 weeks, followed by normoxia for 2 weeks were stained with hematoxylin and eosin. The RV/LV free wall thickness ratio was estimated from these hematoxylin and eosin-stained sections. Values represent the mean ± SD (*n* = 6–10 mice/group). Significant differences between the genotype-matched mice under normoxic and hyperoxic conditions are indicated by *, *p* < 0.05. (ANOVA: Effect: hyperoxia, Interaction: No).

On P28, transthoracic high-resolution echocardiographic studies were performed, and indices of PH including PAT and RVSP were estimated to elucidate the effects of the *AMPKα1* gene, neonatal hyperoxia exposure, and their interactions on PH. Neonatal hyperoxia exposure decreased PAT (Figure 5A–E) and increased the estimated RVSP (Figure 5F) compared with mice exposed to normoxia. These effects of hyperoxia were significantly greater in *AMPKα1^+/−^* mice (PAT: 11.67 ± 1.56 ms; RVSP: 46.20 ± 2.34 mmHg) than in *AMPKα1*^+/+^ mice (PAT: 14.84 ± 0.99 ms; RVSP: 41.44 ± 1.48 mmHg), suggesting that *AMPKα1* deficiency worsens neonatal hyperoxia-induced PH. The heart rate was comparable among all our experimental groups (Figure 5G). Next, we quantified right ventricular hypertrophy (RVH), which is a marker of severe PH. Although Fulton’s index is used to estimate RVH in rodents, the technical challenges associated with estimating this index in neonatal mice can lead to the inaccurate quantification of RVH. Therefore, we used the RV/LV free wall thickness ratio, an alternatively accepted method of quantifying RVH in neonatal mice. Hyperoxia exposure increased the RV/LV free wall thickness ratio; however, *AMPKα1* gene expression did not have an independent effect on RVH in our model (Figure 6).

### 3.4. AMPKα1 Signaling Is Necessary for HPMEC Tubule Formation

**Figure 7:**
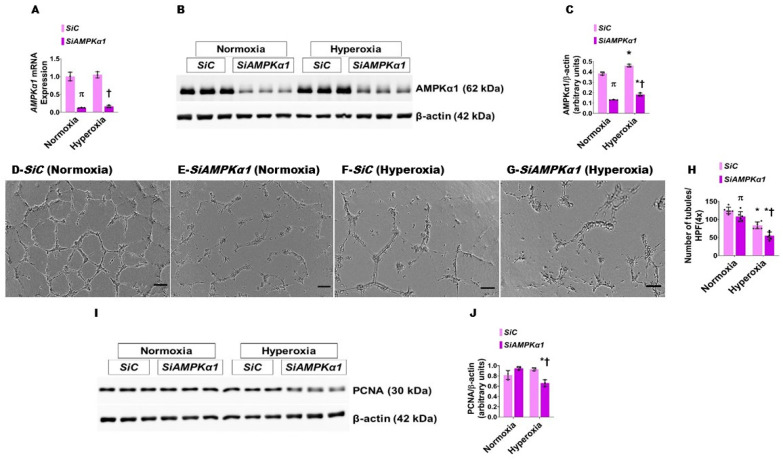
The effect of *AMPKα1* knockdown and hyperoxia on HPMEC tubule formation. HPMECs transfected with control (*SiC*) or *AMPKα1* (*SiAMPKα1*) siRNA and exposed to 21% O_2_ + 5% CO_2_ (normoxia) or 70% O_2_ + 5% CO_2_ (hyperoxia) for 48 h were harvested for gene and protein expression studies and tubule formation assay. (**A**) RT-PCR analyses of *AMPKα1* mRNA levels. (**B**,**C**) Immunoblot determination of *AMPKα1* and β-actin protein levels (**B**) and quantification and normalization of *AMPKα1* band intensities to β-actin (**C**). (**D**–**G**) Representative pictures showing the tubule formation ability of control (**D**,**F**) or *AMPKα1* (**E**,**G**) siRNA transfected cells exposed to normoxia (**D**,**E**) or hyperoxia (**F**,**G**). (**H**) Tubule formation quantitative analyses of control (*SiC*) or *AMPKα1* (*SiAMPKα1*) siRNA-transfected cells. (**I**,**J**) Immunoblot determination of PCNA and β-actin protein levels (**I**) and quantification and normalization of PCNA band intensities to β-actin (**J**). Values are presented as mean ± SD (*n* = 3/group for gene and protein expression studies and *n* = 6/group for tubule formation assay). Significant differences between control- and *AMPKα1*-transfected cells are indicated by π, *p* < 0.05 under normoxic conditions and by †, *p* < 0.05 under hyperoxic conditions. Significant differences between the transfection-matched cells under normoxic and hyperoxic conditions are indicated by *, *p* < 0.05. (ANOVA: *AMPKα1* mRNA expression—Effect: *AMPKα1* transfection, Interaction: No; *AMPKα1* protein expression—Effect: *AMPKα1* transfection and hyperoxia, Interaction: No; Tubule formation—Effect: *AMPKα1* transfection and hyperoxia, Interaction: No; PCNA expression—Effect: *AMPKα1* transfection, Interaction: Yes).

To determine if *AMPKα1* signaling is necessary for lung angiogenesis in human neonates, we used siRNA to knock down *AMPKα1* gene in HPMECs. *AMPKα1* siRNA decreased the mRNA (Figure 7A) and protein (Figure 7B,C) expression of *AMPKα1* in both normoxic and hyperoxic conditions. *AMPKα1* knockdown decreased HPMEC tubule formation in both normoxic and hyperoxic conditions (Figure 7D–H). Importantly, knockdown of this gene significantly decreased HPMEC tubule formation in the hyperoxia group (Hyperoxia: *SiAMPKα1*, 54.83 ± 10.59 vs. *SiC*, 83.67 ± 9.24; *p* < 0.05) (Figure 7F–H). Proliferating cell nuclear antigen (PCNA) plays an important role in cell proliferation, and is often used as an index of cellular proliferation [37]. Whereas hyperoxia exposure did not alter the PCNA protein levels in *AMPKα1*-sufficient cells in our in vitro model, it significantly reduced the PCNA levels in *AMPKα1*-deficient cells (Figure 7I,J), indicating that the *AMPKα1* may regulate angiogenesis under hyperoxic conditions, partly via PCNA.

## 4. Discussion

In this study, we examined the effects of *AMPKα1* knockdown on lung development and PH in a murine BPD model. Furthermore, we performed translational studies using HPMECs to decipher the necessary role of *AMPKα1* in lung angiogenesis. We demonstrate that *AMPKα1* deficiency potentiates hyperoxia-induced neonatal murine lung injury. Additionally, we show that *AMPKα1* signaling is necessary for HPMEC tubule formation.

Lung vascular health maintains alveolar homeostasis and promotes alveolar growth [38,39]. Impaired angiogenesis disrupts alveolarization [40,41,42]. Additionally, reduced growth capacity and abnormal vasoreactivity and extracellular matrix of the lung endothelial cells increases the BPD-PH risk [43,44,45]. Thus, understanding how the lung vascular system homeostasis or health is maintained is pivotal to provide tailored therapies for BPD- PH in preterm infants. Emerging evidence indicates that AMPK promotes vascular health in several organs, including the lungs [21,46,47,48,49,50]. Furthermore, we recently observed that hyperoxia increases pulmonary AMPKα activation in a murine model of BPD-PH [19]. Yadav et al. demonstrated that hyperoxia decreased pulmonary AMPK function in rat pups after 10days of hyperoxia, and the decreased *p*-AMPK levels persisted at P21, 10 days after pups were returned to normoxia [21]. Several other investigators have shown an increase in AMPK activation after shorter exposure to hyperoxia, in human lung fibroblasts and human umbilical vein endothelial cells (HUVECs) [51,52]. However, whether endothelial AMPK, especially the α1 subunit, potentiates or mitigates hyperoxia-induced neonatal lung injury is not well studied, providing a strong premise for our study to clarify its role further.

*Tie2*-driven Cre recombinase was used to decrease the expression of *AMPKα1* in the lung endothelium. Importantly, *Tie2* is also expressed in other cell types, including macrophages and monocytes. Thus, it is possible that some of our results reflect the deficiency of *AMPKα1* in these hematopoietic cells. However, the endothelial cells are significantly enriched with *Tie2*, and *Tie2*-Cre mice are frequently used to study the role of endothelial signaling in lung health and disease [20,53,54]. Furthermore, our hyperoxia model recapitulates the BPD-PH phenotype of infants [26,27]. In alignment with this concept, our hyperoxia-exposed animals displayed alveolar and pulmonary vascular simplification. Significantly, hyperoxia-mediated BPD-PH was potentiated in endothelial *AMPKα1*-deficient mice. Our findings underpin the lung vascular health’s essential role in lung development, i.e., vascular hypothesis [11,55]. Additionally, our results signify the necessary role of endothelial *AMPKα1* in mediating lung angiogenesis, and decreasing neonatal lung disease burden when exposed to a risk factor like hyperoxia. *AMPKα*-deficient mice display increased lung injury when exposed to insults such as lipopolysaccharide [56], particulate matter [57,58], and hemorrhagic shock [59]. Our results add to this existing body of literature, and highlight the protective role of *AMPKα* in lung injury across the rodent life span. We also demonstrate that the necessary role of *AMPKα1* extends beyond murine lungs. Consistent with other studies [15,46,50,60], we show that *AMPKα* deficiency decreases human lung endothelial cell angiogenesis. PCNA plays a major role in DNA replication, and is frequently used as a marker of cellular proliferation [61]. Thus, our data indicate that *AMPKα1* regulates HPMEC tubule formation in hyperoxic conditions partly via PCNA-dependent mechanisms. Interestingly, we noted increased *AMPKα2* protein expression in endothelial cell *AMPKα1*-deficient mice. Pulmonary hypertension is a significant morbidity of endothelial cell *AMPKα2* deficient mice [49]. Therefore, the increase in the *AMPKα2* protein seen in our model may be a compensatory response to maintain the total endothelial AMPKα function. Nevertheless, the compensatory increase in *AMPKα2* protein was insufficient to rescue our AMPKα1-deficient mice from hyperoxia-mediated lung injury. We also determined the effects of endothelial *AMPKα1* deficiency on pulmonary vascular remodeling and function. Although the gold standard diagnostic test for PH is cardiac catheterization, noninvasive echocardiography has been commonly used to delineate cardiac structure and function in small animals, because of its technical feasibility and reliability for determining experimental PH. Furthermore, the RV systolic time intervals, such as PAT determined by echocardiography, correlate with the PA pressure measured by cardiac catheterization [26,33,62]. PAT correlates inversely, while RVSP correlates directly with the pulmonary artery pressure [62,63,64]. Thus, our findings reinforce the fact that exposure to moderate hyperoxia for a prolonged duration induces PH in neonatal mice. Similarly, hyperoxia induced pulmonary vascular remodeling, another PH biomarker, in our model. Our results also demonstrate that endothelial *AMPKα1* deficiency potentiates hyperoxia-induced PH, signifying the necessary role of endothelial *AMPKα1* to mitigate experimental PH. Studies in adult rodents have demonstrated the necessary and sufficient function of *AMPK* in preventing and mitigating PH through several mechanisms, including the inhibition of autophagy and proliferation of pulmonary artery smooth muscle cells [65,66,67]. Our study validates these findings and extends the potential protective effect of endothelial *AMPKα1* for BPD-PH. We did not observe a statistically significant independent effect of *AMPKα1* on hyperoxia-mediated effect on the RV/LV ratio. One possibility for this observation is that we used mice that were partially deficient in *AMPKα1*, rather than those that completely lacked *AMPKα1*.

The strengths of our study include the use of: (1) a rigorous genetic technique determining endothelial *AMPKα1′*s role in developmental lung injury; (2) high-resolution echocardiography to elucidate endothelial *AMPKα1′*s effects on cardio-pulmonary function; and (3) human neonatal lung endothelial cells determining *AMPKα1′s* role in human lung angiogenesis, increasing the clinical relevance of our study. Despite these strengths, our study has a few limitations that we will address later. We did not elucidate the: (1) sex-specific effects of endothelial *AMPKα1* deficiency; (2) impact of hyperoxia or endothelial *AMPKα1* deficiency on lung function; (3) impact of *AMPKα1* activation on hyperoxia-induced experimental BPD-PH; and (4) precise molecular mechanisms through which endothelial *AMPKα1* deficiency potentiates neonatal hyperoxic lung injury.

**Figure 8:**
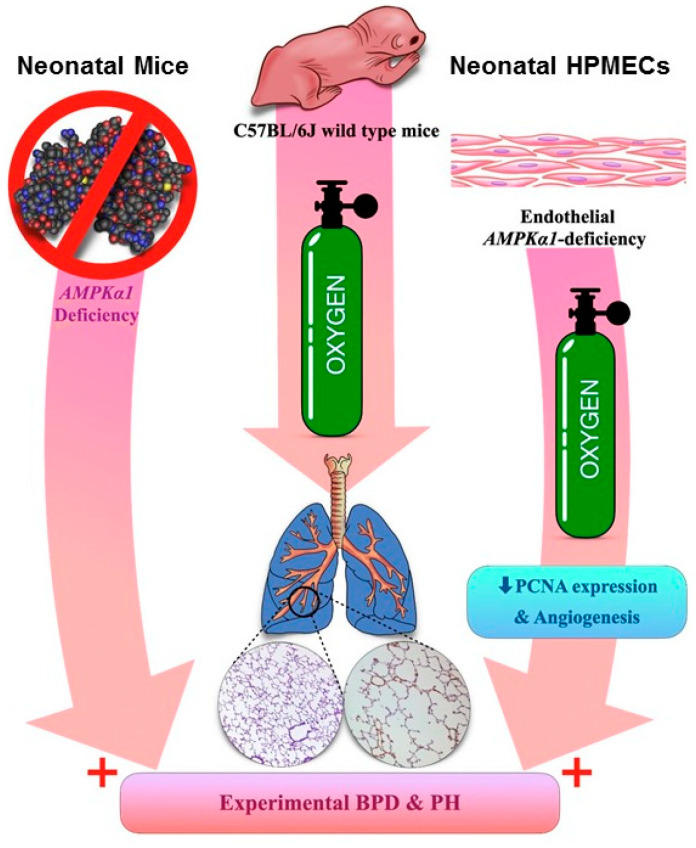
Schema of our overall findings: *AMPKα1*—adenosine monophosphate-activated protein kinase (AMPK) α1; HPMECs—human pulmonary microvascular endothelial cells; PCNA—proliferating cell nuclear antigen; BPD—bronchopulmonary dysplasia; PH—pulmonary hypertension.

In summary, we show that *Tie2*-Cre-mediated endothelial *AMPKα1* deficiency potentiates hyperoxia-induced experimental BPD-PH in mice. Furthermore, we show that *AMPKα1* is required for angiogenesis and PCNA expression in human neonatal lung endothelial cells exposed to hyperoxia, which we speculate are some of the mechanisms through which *AMPKα1* regulates experimental BPD-PH (Figure 8). To the best of our knowledge, this is the first study that elucidates the essential role of endothelial *AMPKα1* in hyperoxia-mediated experimental BPD-PH, and emphasizes that *AMPKα1* is a potential therapeutic target for BPD infants who develop PH.

## 5. Conclusions

*AMPKα1* signaling is necessary to mitigate hyperoxia-induced BPD and PH in neonatal mice and promote angiogenesis in neonatal HPMECs.

## Data Availability

The data presented in this study are available in the article.
